# The NPGS Sudan sorghum germplasm collection reveals a novel cluster of R genes associated with rust resistant

**DOI:** 10.1002/tpg2.70113

**Published:** 2025-09-15

**Authors:** Hugo E. Cuevas, Louis K. Prom

**Affiliations:** ^1^ USDA‐ARS Tropical Agriculture Research Station Mayagüez Puerto Rico; ^2^ USDA‐ARS Southern Plains Agriculture Research Center College Station Texas USA

## Abstract

Leaf rust, caused by the obligate fungal pathogen *Puccinia purpurea*, poses a serious threat to sorghum [*Sorghum bicolor* (L.) Moench] production leading to significant yield losses and undermining its values as renewable fuel crop. In this study, the United States Department of Agriculture—Agriculture Research Service, National Plant Germplasm System (NPGS) Sudan core collection was evaluated for rust‐resistant response across four tropical environments. The analysis identified 18 accessions with rust resistant, among which four accessions (PI 568621, PI 569393, PI 570548, and PI 570974) consistently showed no rust pustules across all environments. Genome‐wide association analysis led to the identification of a 57 kbp genomic region on chromosome 8 that encompasses a cluster of five homologous R genes. The resequencing analysis of the first exon from one candidate gene (*Sobic.008G178200*) found 61 point mutations that generate seven haplotypes. The high homology of these five genes and seven haplotypes indicates that this cluster might be acting as a single locus (*Rp2*) against *P. purpurea*. Comparative genome analysis found that the orthologs of *Rp2* locus in maize (*Zm00001d023311*) are associated with the resistant response to *Puccinia polysora*, the causal agent of southern corn rust and in rice (*Os12G29690*), with resistance to the brown planthopper (*Nilaparvata lugens*). The introgression of the *Rp2* locus into elite varieties or the inclusion of top‐performing Sudanese tropical accessions in pre‐breeding germplasm can accelerate the development of improved sorghum germplasm with durable rust resistant.

## Core Ideas

- The *Sorghum bicolor* National Plant Germplasm System (NPGS) Sudan core collection contains new sources of leaf rust resistant.
- The population structure of the NPGS Sudan core collection is associated with the frequency of rust resistant.
- Genome‐wide association analysis revealed a new rust resistance locus (*Rp2*) on chromosome 8.
- The rust resistance locus (*Rp2*) is a 57 kbp genomic region that encloses a cluster of five homologous R genes.
- Orthologs of *Rp2* locus in maize and rice are associated with fungal disease and insect resistant.

## Plain Language Summary

The National Plant Germplasm System (NPGS) Sudan sorghum core collection of the United States Department of Agriculture is an important source of genetic diversity for the development of new sorghum varieties. Therefore, a total of 268 accessions from this core collection were evaluated for leaf rust (*Puccinia purpurea*) resistant across four tropical environments. Eighteen accessions with leaf resistant were identified, of which four consistently showed no rust symptoms across all environments. Genome‐wide association analysis identified a cluster of R genes associated with the observe resistance response. These NPGS Sudanese accessions are useful in a pre‐breeding germplasm program to develop new improved germplasm with novel sources of rust resistant.

**Abbreviations**
ANOVA: analysis of variance
BLAST: Basic Local Alignment Search Tool
farmCPU: fixed and random model Circulating Probability Unification
GBS: genotyping‐by‐sequencing
GWAS: genome‐wide association studies
LD: linkage disequilibrium
LRR: leucine rich repeats
NBS: nucleotide binding site
NPGS: National Plant Germplasm System
SAP: sorghum association panel
SNP: single nucleotide polymorphism
TARS: Tropical Agricultural Research Station
USDA‐ARS: United States Department of Agriculture—Agriculture Research Service

## 1 INTRODUCTION

Crop improvement depends on the adequate knowledge of its existing genetic diversity. Ex situ germplasm collection (i.e., gene banks) is the primary source of genetic diversity of plant breeding programs (Swarup et al., 2021). Sorghum [*Sorghum bicolor* (L.) Moench], the fifth most important cereal of the world, is a crop with rich diversity that was domesticated in the dry northeast region of Africa more than 5000 years ago (Smith & Frederiksen, 2000). Nevertheless, multiple independent re‐domestication events occurred in the western and southern regions of Africa resulting in adaptation to different environments. These re‐domestication event resulted in five major races (kafir, durra, caudatum, guinea, and bicolor) differentiated by inflorescence type (Harlan & Dewet, 1972). Most of this sorghum genetic diversity is being preserved across multiple germplasm collections located in the United States, India, Ethiopia, Australia, China, and Nigeria. The largest sorghum germplasm collection is maintained by the United States Department of Agriculture—Agriculture Research Service (USDA‐ARS) National Plant Germplasm System (NPGS) and includes >40,000 accessions from 114 countries. Interestingly, 7217 and 4088 accessions are originally from the center of domestication located across Ethiopia and Sudan, respectively. The large size of most sorghum germplasm collections has limited the phenotypic characterization and hence encouraged the use of high‐throughput phenotyping and genotyping platforms that lead to the identification of the most valuable accessions.

The establishment of representative subsets that capture most of the genetic diversity of a germplasm collection (i.e., core collection; Brown, 1989) is the first step to hasten the identification of valuable accessions. In this regard, a core collection of 3011 accessions was assembled for the NPGS sorghum germplasm collection (Dahlberg et al., 2004). However, the screening of this core collection is constrained by the tropical short‐day length of most accessions which require less than 12 h of daylight and warm temperatures to reach full maturity (i.e., photoperiod sensitivity). To better optimize strategies and resources for preserving and utilizing NPGS genetic diversity in breeding programs, subsets from Ethiopia, Sudan, Nigeria, Niger, and Senegal origin have been genetically characterized using genotyping‐by‐sequencing (GBS; Elshire et al., 2011). Later, these large‐scale genotyping have been used for the genomic dissection of agronomically important traits (Cuevas & Prom, 2020; Cuevas et al., 2017; Faye et al., 2019; Maina et al., 2018; Olatoye et al., 2018). For instance, population structure analyses and phenotypic characterization of these countries’ subsets for agronomic traits, anthracnose, and grain mold resistance led to the identification of valuable accessions for breeding programs. Further, genome‐wide association studies (GWASs) had uncovered novel disease resistance loci that led to the pinpointing of genes of agronomic importance (Cuevas & Prom, 2024; Cuevas et al., 2019; Faye et al., 2019). These studies have provided evidence that the NPGS tropical germplasm contains novel alleles and genetic diversity currently absent in sorghum breeding programs. In this regard, the sorghum association panel (SAP) comprised of 377 temperate‐adapted accessions, representing most of the genetic diversity of US breeding programs (Casa et al., 2008; Morris et al., 2013), could be used as a repository for introgression of novel sources of variation from NPGS tropical germplasm. Certainly, sorghum improvement depends on the allele mining of economically important traits within the genetic diversity of the NPGS sorghum collection.

Sorghum is a C_4_ grass with drought tolerance and is mainly grown as a staple cereal crop in arid and semi‐arid regions of the world (Borrell et al., 2024). It is also used as a source of food, feed, fodder, and biomass, which is for use in renewable fuel production (Visarada & Aruna, 2021). The broad range of end uses of sorghum has spread its cultivation to a wide array of climatic conditions. Simultaneously, the expansion of sorghum cultivation has resulted in the challenge of multiple abiotic and biotic stresses that reduce crop yield. The production regions with warm and humid climates are mainly affected by fungal diseases that affect the whole plant (leaves, stem, and grain) (Little & Perumal, 2019). Leaf rust, caused by the obligate fungal pathogen *Puccinia purpurea* Cooke, causes significant yield reduction if it occurs during early stages of plant development (White et al., 2012) and exacerbates the occurrence of other diseases, such as anthracnose (*Colletotrichum sublineola*) (Murali Mohan et al., 2010). The disease is characterized by the production of small, brown uredial pustules that arise from above the leaf surface (Little & Perumal, 2019). Deploying resistant cultivars is the most cost‐effective management strategy to control rust disease.

The inheritance and precise molecular mechanisms of rust resistant response in sorghum are not well understood. Moreover, the limited number of known rust resistant accessions and locus in breeding programs could lead to the collapse of resistant sources. For instance, the *Pu* resistance locus identified in sweet sorghum cultivars MN 960 and QL42 is located on chromosome 8 (Miller & Cruzado, 1969; Tao et al., 1998). Comparative mapping analysis found that the genomic region encompassing the maize [*Zea mays* (L.)] *Rp1‐D* and wheat (*Triticum aestivum*) *Lr* rust resistance genes is homologous with the *Pu* locus (Mace & Jordan, 2010; McIntyre et al., 2005) demonstrating gene conservation function after divergency. Later, a GWAS using diversity panel and biparental mapping populations revealed the polygenic nature of the rust‐resistant response (Murali Mohan et al., 2010; Upadhyaya et al., 2013). These studies led to the identification of multiple genomic regions associated with rust resistance response, including a genomic region on chromosome 6 that colocalizes with the plant color locus (*Plcor*; Klein et al., 2001; Srinivas et al., 2009). Candidate gene analysis within this quantitative trait locus (QTL) highlighted genes involved in pathogen recognition (i.e., resistance [R] genes) and plant immune system suggesting that each resistant source might have a unique defensive mechanism.

The identification of new rust‐resistant sources and the elucidation of their molecular mechanisms are crucial to achieve sustainable control of the disease. In this study, we evaluated the NPGS Sudan core collection to (1) identify new rust‐resistant sources that could be used to increase the genetic diversity of breeding programs and (2) conduct GWAS to identify novel resistance loci that enhance our understanding of the underlying defense mechanisms and to provide single nucleotide polymorphism (SNP) markers for marker‐assisted selection. These results will benefit sorghum breeding programs worldwide focused on the development of new rust‐resistant varieties and may facilitate the pyramiding of multiple resistance genes.

**Core Ideas**

- The *Sorghum bicolor* National Plant Germplasm System (NPGS) Sudan core collection contains new sources of leaf rust resistant.
- The population structure of the NPGS Sudan core collection is associated with the frequency of rust resistant.
- Genome‐wide association analysis revealed a new rust resistance locus (*Rp2*) on chromosome 8.
- The rust resistance locus (*Rp2*) is a 57 kbp genomic region that encloses a cluster of five homologous R genes.
- Orthologs of *Rp2* locus in maize and rice are associated with fungal disease and insect resistant.

## 2 MATERIALS AND METHODS

### 2.1 Germplasm and field experiment

A total of 268 accessions from the NPGS Sudan core collection (Dahlberg et al., 2004) were evaluated for rust resistant (Table S1). The core set and susceptible (PI 609251, RTx2911 [PI 607931]) and resistant (BTx623 [PI 564163], Sureño [PI 561472]) accessions were planted on research farms of the USDA‐ARS, Tropical Agricultural Research Station (TARS) at Isabela (18°28′18.6″ N 67°02′37.1″ W) and Mayaguez (18°12′41.5″ N 67°08′08.0″ W), Puerto Rico, in 2014 and 2016, respectively. At both locations, a completely randomized design was used with plots measuring 1.8 m in length and 0.9 m between rows. Plants were maintained using standard management practices such as weed control with mechanical tillage and hand hoeing, and aerial watering (raingun irrigation) was supplied in the absence of rainfall. This phenotypic evaluation was used to select a subset of 24 rust‐resistant accessions at both locations, 27 resistant at either location, and 36 accessions that showed a broader spectrum of susceptibility to rust to be evaluated for two additional years (2020 and 2021). This subset of 87 Sudanese accessions together with susceptible (PI 609251, RTx2911 [PI 607931]), and resistant (BTx623 [PI 564163], Sureño [PI 561472]) checks were planted on the research farms of TARS at Isabela, Puerto Rico, in randomized complete block design consisting of two blocks with plots of 1.8 m in length and 0.9 m between rows.

### 2.2 Rust resistant response

Rust‐resistant response of all accessions was evaluated at the hard dough stage of plant development (i.e., ≥30 days post flowering). A total of 5–10 plants per plot were visually inspected, and the most infected plant was used for disease assessment based on the rust severity of leaves from the middle of the stalk to the top of the plant. Rust severity was based on a scale of 1–5; where 1 = no rust pustules, leaves free of the disease; 2 = 1%–10% leaf area infected; 3 = 11%–40% leaf area infected; 4 = 41%–65% leaf area infected; 5 = 66%–100% leaf area infected (M. L. Wang et al., 2006). This scale was further used to classify accessions into those resistant (<2.0), moderately resistant (≥2.0 but <3.0), and susceptible (≥3.0) to rust.

### 2.3 Phenotypic analysis

The rust‐resistant responses of the 268 accessions from the NPGS Sudan collection were subjected to analysis of variance (ANOVA) using the PROC MIXED COVTEST METHOD = TYPE3 procedure of SAS (SAS Institute). The locations and accessions were considered fixed and random effects, respectively. Rust‐resistant response was estimated based on the average across locations. Subsequently, the data of the four experiments that include the subset of 87 accessions were combined to perform ANOVA using the PROC MIXED COVTEST METHOD = TYPE3 procedure of SAS. The year and accessions were considered fixed effects, and blocks were treated as random effects. Rust‐resistant response for these accessions was estimated based on the average across years.

### 2.4 GBS and population structure

The genomic information of the 268 accessions from the NPGS Sorghum germplasm collection is a community resource (Cuevas & Prom, 2020) available at the National Center for Biotechnology Information (NCBI) sequence read archive (SRP222368). This previous SNPs call based on the BTx623 reference genome v.3 identified a total of 183,184 SNPs, of which a subset of 5366 unlinked SNPs was used to reveal the existence of five ancestral populations (Cuevas & Prom, 2020). This SNP call and population structure analysis was used herein for phylogenetic and GWAS of rust resistant.

### 2.5 Cluster analysis

The NPGS Sudanese rust‐resistant accessions were subjected to clustering analysis using the 5366 unlinked SNPs. A phylogenetic tree was created using the maximum likelihood method with an optimal model selected with ModelFinder (Kalyaanamoorthy et al., 2017) as implemented in IQ‐TREE 2 software (Minh et al., 2020). The ultrafast bootstrapping method (Hoang et al., 2018) was used to determine branch support. The phylogenetic tree was visualized using Interactive Tree of Life (Letunic & Bork, 2011).

The frequency of rust‐resistant response observed among the five populations present in the NPGS Sudan germplasm collection (Cuevas & Prom, 2020) was also compared using the Tukey–Kramer post hoc test at the *p* ≤ 0.05 level of significance. In addition, the frequency of rust resistant (≤2.0) and susceptibility (>2.0) in each population and the entire NPGS Sudan germplasm collection was determined. Later, *χ* ^2^ tests were performed to determine if rust‐resistant frequency in each population deviated from the expected rust‐resistant frequency observed in the entire NPGS Sudan germplasm collection. Segregation distortion against the expected ratio (*χ* ^2^ *p* < 0.01) was considered a signature of selection for the resistant/susceptible rust response.

### 2.6 Genome‐wide association study

The GWAS for rust resistant was conducted with the fixed and random model Circulating Probability Unification (farmCPU; Liu et al., 2016) model using the GAPIT v.3 (J. Wang & Zhang, 2021) package in the R v.4.4.1 software. To reduce spurious association, the first three components of the principal components analysis were included as covariants, while the farmCPU model substitutes the kinship matrix with a set of markers within the associated genomic region. Significant associations were determined using the false discovery rate (Benjamini & Hochberg, 1995) using a cutoff of *p* ≤ 0.01 as implemented in GAPIT. Manhattan and log quantile‐quantile *p*‐value plots were visualized using the R package CMplot (Yin, 2022). The associated genomic regions were delimited based on the linkage disequilibrium (LD) analysis with the associated SNPs using the block function of PLINK (Purcell et al., 2007). This genomic region was examined for candidate genes using the annotation of the BTx623 v.3.1.1 reference genome.

### 2.7 Allelic diversity in candidate gene

The candidate gene *Sobic.008G178200* was partially sequenced in a subset of 14 Sudanese accessions (two resistant [<2.0], five moderately resistant [≥2.0 but <3.0], and seven susceptible [≥3.0]). This set of primers (Forward: GATGGTCAGCGAAGAGCAG; Reverse: TGCCTGCTT GTTACCACAAC) specific for the first exon of *Sobic.008G178200* was designed using Primer3 v.4.1 (Untergasser et al., 2012). The amplicon was sequenced via BigDye terminator chemistry (North Carolina State University, Genomic Science Laboratory), and the sequence chromatograms were examined using SEQUENCHER (version 5.4.6; GENECODES). Haplotypes were visually identified and amino acid sequences were aligned in Clustal W (Larkin et al., 2007). Phylogenetic analysis among haplotypes was conducted in IQ‐TREE 2.2.2.6 software (Minh et al., 2020) using the maximum likelihood method and the tree was selected with ModelFinder (Kalyaanamoorthy et al., 2017). The ultrafast bootstrapping (Hoang et al., 2018) method as implemented in IQ‐TREE 2 to determine branching support values based on 1000 repetitions. The phylogenetic tree was visualized using Interactive Tree of Life (Letunic & Bork, 2011).

### 2.8 Comparative gene analysis

The orthologs in maize and rice of the R gene *Sobic.008G178200* were identified using the Basic Local Alignment Search Tool (BLAST) against the annotation of B73 v.4 and Nipponbare v.7 reference genomes, respectively. In addition, the orthologs in rice and sorghum of the maize rust resistance gene *Rp1‐D* (Zm00001d023325) (Collins et al., 1999) were identified using the BLAST against the annotation of Nipponbare v.7 and BTx623 v.3.1.1 reference genomes, respectively. Amino acid sequences of the genes were aligned using CLUSTAL W (Larkin et al., 2007). Phylogenetic analysis among amino acids sequences was conducted in IQ‐TREE 2 software (Minh et al., 2020) using the maximum likelihood method and select the tree with ModelFinder (Kalyaanamoorthy et al., 2017). Ultrafast bootstrapping (Hoang et al., 2018) method as implemented in IQ‐TREE 2 was used to determine branching support values based on 1000 repetitions. The phylogenetic tree was visualized using Interactive Tree of Life (Letunic & Bork, 2011).

## 3 RESULTS

### 3.1 Rust resistant in NPGS Sudan germplasm collection

The rust‐resistant response of the NPGS Sudan core collection at Isabela and Mayaguez, Puerto Rico found that 25 accessions were resistant across locations, while 26 accessions showed a variable resistant response. Most of the accessions were highly susceptible to rust (190 accessions with score ≥ 3.0) with an average infection rating of 3.51 ± 0.07. The 4‐year evaluation of the subset determined that 18 accessions showed rust resistant (<2.0; Table S1) across years and locations. We observed that five accessions (PI 570072, PI 569439, PI 569876, PI 569026, and PI 568990) with a resistant response in Isabela were susceptible against pathotypes from Mayaguez. A total of four accessions (PI 568621, PI 569393, PI 570548, and PI 570974) were consistently resistant across years and locations without the development of rust pustules on the leaves (score = 1.0).

**FIGURE 1:**
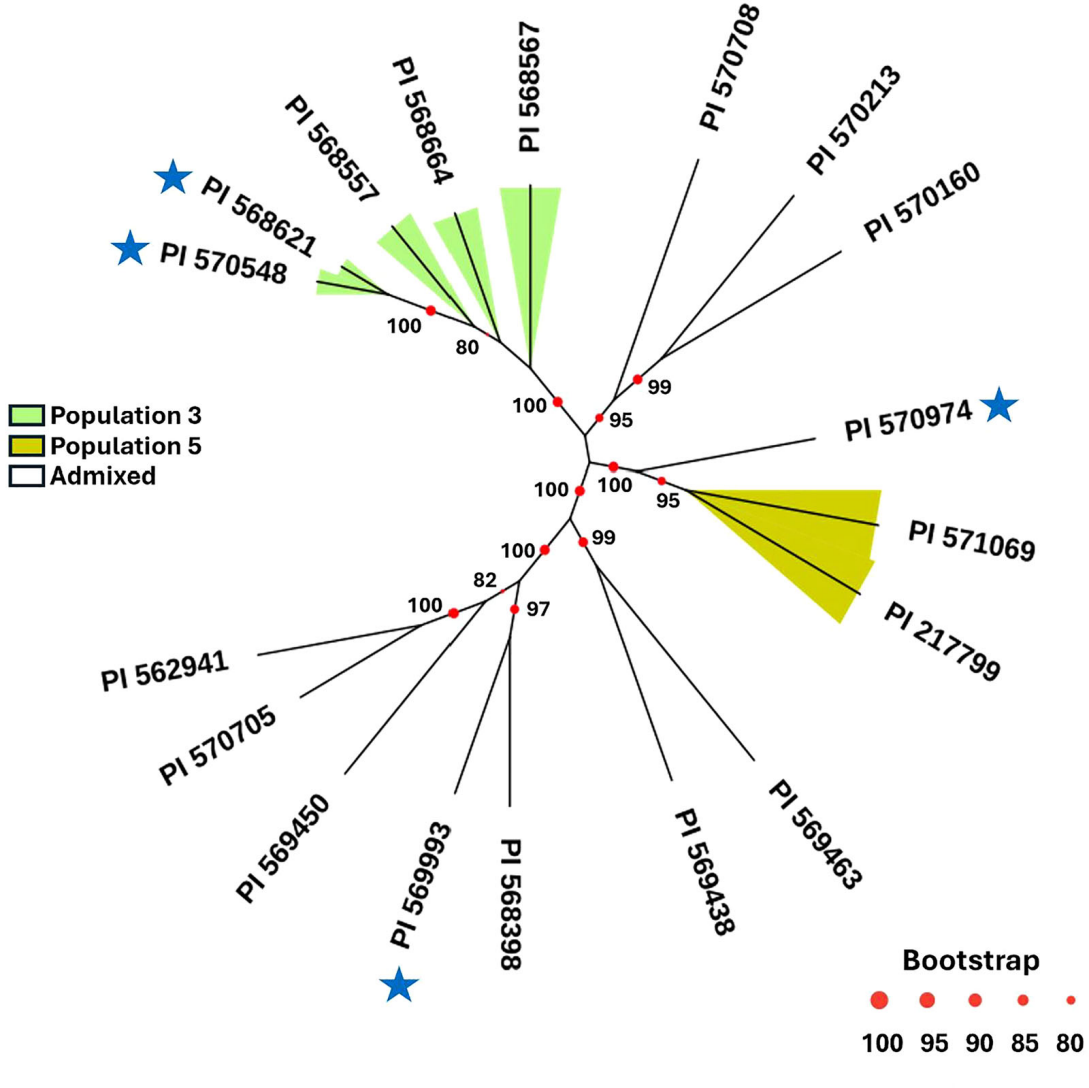
Maximum‐likelihood tree with 1000 bootstrap replicates among 18 rust‐resistant accessions from the National Plant Germplasm System (NPGS) Sudan core collection. Colored branches represent accessions belonging to the five populations present in the NPGS Sudan core collection according to Cuevas and Prom (2020). Bootstrap values >80 are marked on each node with red circle. Accessions that show no rust pustules across all environments are identified with a blue star.

The distribution and genetic relationship among rust‐resistant accessions in the NPGS Sudan core collection suggested the presences of a limited number of resistance sources. The genetic diversity of the NPGS Sudan core collection was divided into five ancestral populations and one admixed group (Cuevas & Prom, 2020). The 18 rust‐resistant accessions were distributed among population 3 (five accessions), 5 (two accessions), and the admixed group (11 accessions) (Table 1). The analysis found signatures of selection for rust resistant in population 3 (*χ* ^2^ = 17.0; *p* < 0.0001), which includes a higher frequency of resistant accessions and the lowest disease score average. In contrast, the accessions belonging to population 1 were highly susceptible to rust (4.13 ± 0.60). The frequency of rust‐resistant accessions in population 5 and the admixed group do not deviate from that observed in the whole NPGS Sudan core collection. To determine the most likely number of resistance sources present in the NPGS Sudan core collection, a phylogenetic analysis among the 18 resistant accessions was conducted. The maximum likelihood tree showed a close genetic relationship between some resistant accessions and clusters according to the population structure of the NPGS Sudan core collection (Figure 1). The resistant accessions from populations 3 and 5 constitute two branches of the tree suggesting the resistance mechanism might be similar within population (i.e., identical by descent). The resistant accessions in the admixed group could be divided into three groups based on their admixture coefficient and phylogenetic relationship. We observed that the genome of seven resistant accessions (PI 562941, PI 570705, PI 569450, PI 569993, PI 568398, PI 569438, and PI 569463) are an admixture of populations 3 and 2 (ancestry coefficient of both populations is >0.74), while another three accessions (PI 570160, PI 570213, and PI 570708) are also an admixture of populations 3 and 5 (ancestry coefficient of both populations >0.75). The other accession (PI 570974) is an admixture of populations 5 and 2 (ancestry coefficient of both populations is 0.83). These results suggest that the rust‐resistant response observed in the NPGS Sudan core collection should be determined by two sources originating in populations 3 and 5.

**TABLE 1:** Rust severity in the National Plant Germplasm System (NPGS) Sudan core collection.

NPGS Sudan core set^a^	*n* ^b^	Means ± SD^c^	Resistant^d^	Susceptible	*χ* ^2^ test^e^
Population 1	12	4.13 ± 0.60b	0	12	0.35
Population 2	40	3.48 ± 0.94ab	0	40	0.09
Population 3	17	3.22 ± 1.56a	5	12	0.00
Population 4	19	3.62 ± 0.85ab	0	19	0.24
Population 5	59	3.84 ± 0.96ab	2	57	0.37
Admixed	121	3.32 ± 1.20ab	11	110	0.30

^a^ Population structure analysis according to Cuevas and Prom (2020).

^b^ *n* refers to the number of accessions.

^c^ Means and standard deviation (SD) of rust‐resistant response based on 1–5 scale according to Wang et al. (2006). Means and SDs with the same letter are not significantly different based on least‐significance difference test at the 5% level of significance.

^d^ Accessions with rust severity <2.0.

^e^ Chi‐square test for observe rust‐resistant frequency against the expected based on the frequency of the NPGS Sudan collection.

### 3.2 Genome‐wide association analysis and candidate genes

**FIGURE 2:**
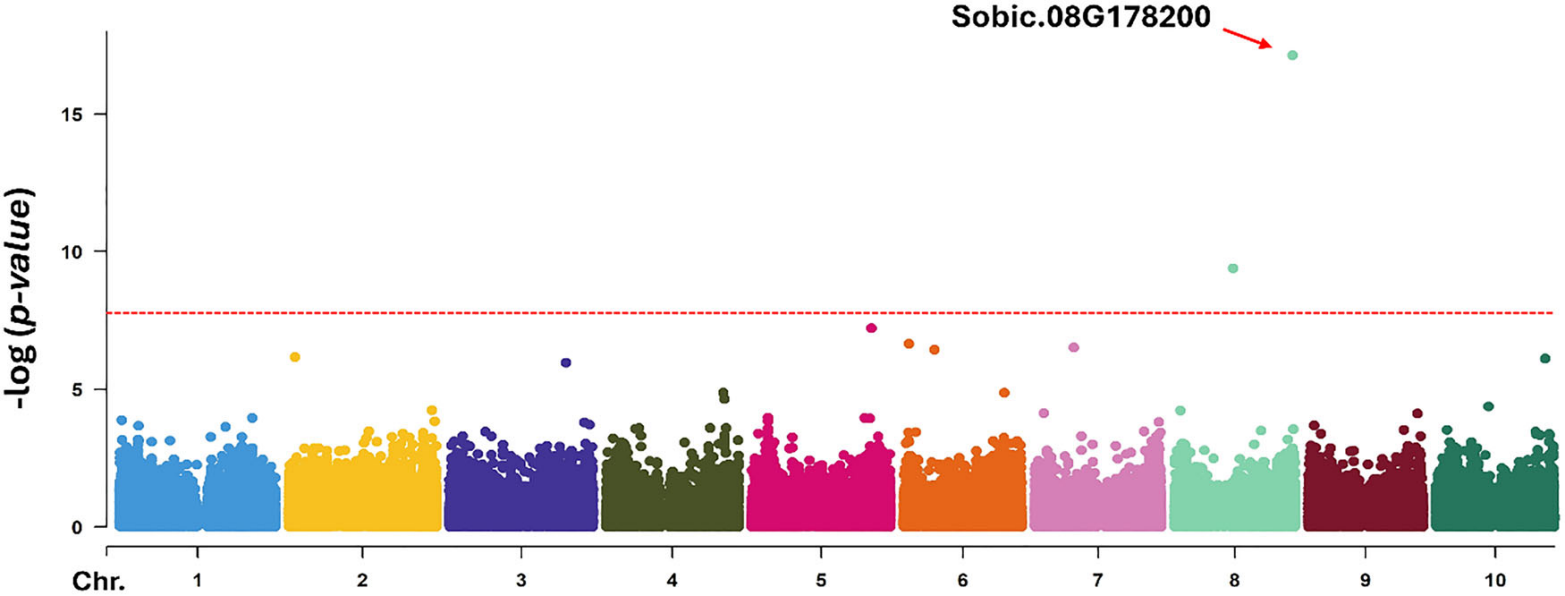
Genome‐wide association analysis for rust resistant in the National Plant Germplasm System (NPGS) Sudan Core collection. Manhattan plots for the fixed and random model Circulating Probability Unification (farmCPU) regression analysis with 187,580 SNPs.

The genome‐wide association analysis for rust resistan led to the identification of two genomic regions in chromosome 8 (Figure 2). The SNP S8_61201912 [−log (*p*‐value) = 17.13] represents an LD genomic region of 6.5 kbp (Ch8: 61,195,478–61,201,929) with a negative additive effect (−0.69) and encloses three putative genes (*Sobic.008G178000*, *Sobic.008G178100*, and *Sobic.008G178200*). We found that *Sobic.008G178200* has two domains that encode a leucine rich repeats (LRR) and nucleotide binding site (NBS), which are both related to disease resistance genes. Remarkably, one homolog of *Sobic.008G178200* was found downstream (*Sobic.008G177900*; 97% identity) and another three with 97%, 85%, and 88% of similarity were upstream (*Sobic.008G178300*, *Sobic.008G178500*, and *Sobic.008G178600*, respectively). These five genes are located within a 57 kbp genomic region that includes seven other genes. The SNP S8_30380270 [−log (*p*‐value) = 9.38; Figure 2] represents an LD genomic region of 6.3 kbp with the nearby SNP S8_30373911. This block is in an intergenic region, which is in linkage equilibrium with the two nearest SNPs located 191 and 60 kbp downstream and upstream, respectively. This locus exhibited a positive additive effect because the allele in minor frequency was associated with susceptibility to rust (i.e., increased the rust severity of leaves by 0.45 on the 1–5 scale).

To assess the effects of the main locus (SNP S8_61201912) within the NPGS Sudan population structure, an independent general regression model was applied to each population (Table 2). Allelic variation was observed only within populations 3, 4, 5 and the admixed group. The frequency of the resistance allele in population 3 (0.31) was higher than that observed in the other populations (0.05–0.07). The variation in the additive effect of the resistance allele ranged from −0.63 (admixed group) to −1.40 (population 3), indicating differential selection pressures among populations.

**TABLE 2:** Allele frequency distribution in the National Plant Germplasm System (NPGS) Sudan core collection for the rust resistance locus S8_61201912 detected through genome‐wide association analysis.

		Ch8: 61201912		General linear model^a^	
NPGS Sudan^b^	*n*	C	G^c^	*p*‐value	Additive effect
Population 1	12	1.00	0.00	n/a	n/a
Population 2	40	1.00	0.00	n/a	n/a
Population 3	16	0.69	0.31	6.13E‐06	−1.40
Population 4	19	0.95	0.05	4.80E‐02	−0.85
Population 5	58	0.94	0.06	5.00E‐03	−0.76
Admixed	115	0.92	0.06	8.00E‐03	−0.63
Overall^d^	260	0.93	0.07	7.46E‐18	−0.69

^a^ Association analysis based on general linear model (GLM) regression. Populations with no‐allelic variation were considered not applicable (n/a) for GLM regression.

^b^ Population structure analysis according to Cuevas and Prom (2020).

^c^ Rust‐resistance allele.

^d^ Association analysis in the overall is based on random model Circulating Probability Unification (farmCPU) regression.

### 3.3 Allelic diversity

**FIGURE 3:**
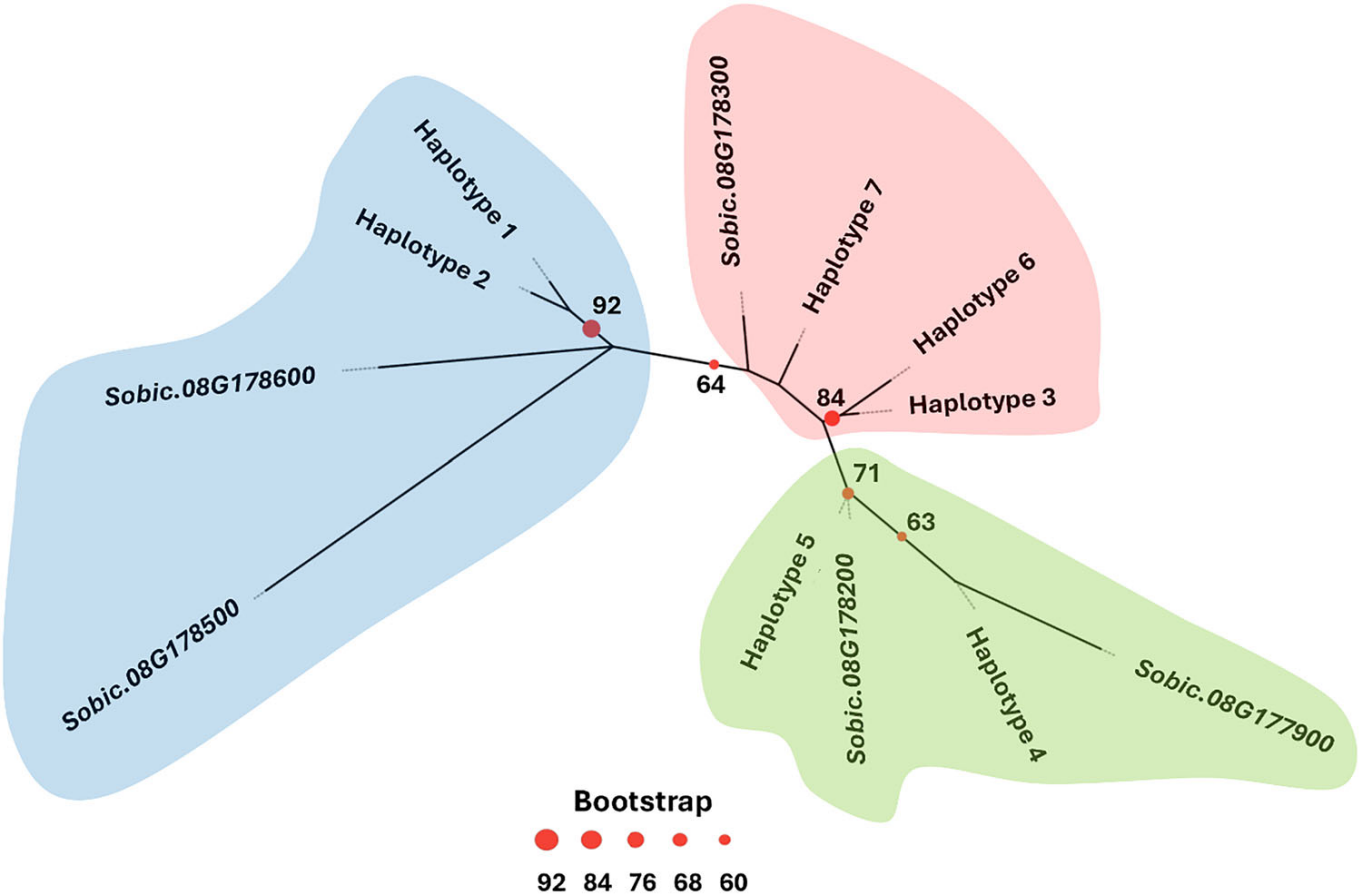
Maximum‐likelihood tree with 1000 bootstrap replicates among the five R genes present in the *Rp2* locus and seven haplotypes found in the first exon of the gene *Sobic.008G178200*. Bootstrap values > 60 are marked on each node with red circle. Blue, red, and green circles highlight the three main genetically related groups of haplotypes.

The re‐sequencing analysis of the 550 bp genomic region from *Sobic.008G178200* found 70 putative SNPs, of which 61 SNPs were located within the protein coding region of exon 1 (Table S2). These 61 SNPs generated seven haplotypes (referered to hereinafter as Hap.1 to Hap.7) that also encode different protein structures. The 162 amino acid alignment for exon 1 found that these SNPs lead to 31 amino acid changes across haplotypes, of which 8, 10, and 13 were conserved, semi‐conserved, and non‐conserved substitutions, respectively. A phylogenetic analysis of the haplotype's genetic sequences clustered Hap.1 and Hap.2 separately from other haplotypes (Figure 3). The genetic differences between Hap.1 and Hap.2 were based on eight SNPs and both haplotypes have genetic similarities with the putative R genes *Sobic.008G178500* and *Sobic.008G178600*. Likewise, Hap.4 and Hap.5 were genetically related and have genetic similarities with the putative R genes *Sobic.008G177900* and *Sobic.008G178200*. We also observed that, Hap.3, Hap.6, and Hap.7 share higher genetic similarities and cluster near to *Sobic.008G178300*. Therefore, these seven haplotypes could be grouped into three genetic groups. The haplotype genetic diversity found in the exon 1 of *Sobic.008G178200* and its genetic relationship with nearby R genes indicated these five genes act as a single locus against *P. purpurea* named hereafter as *Rp2*.

### 3.4 Comparative gene analysis

**FIGURE 4:**
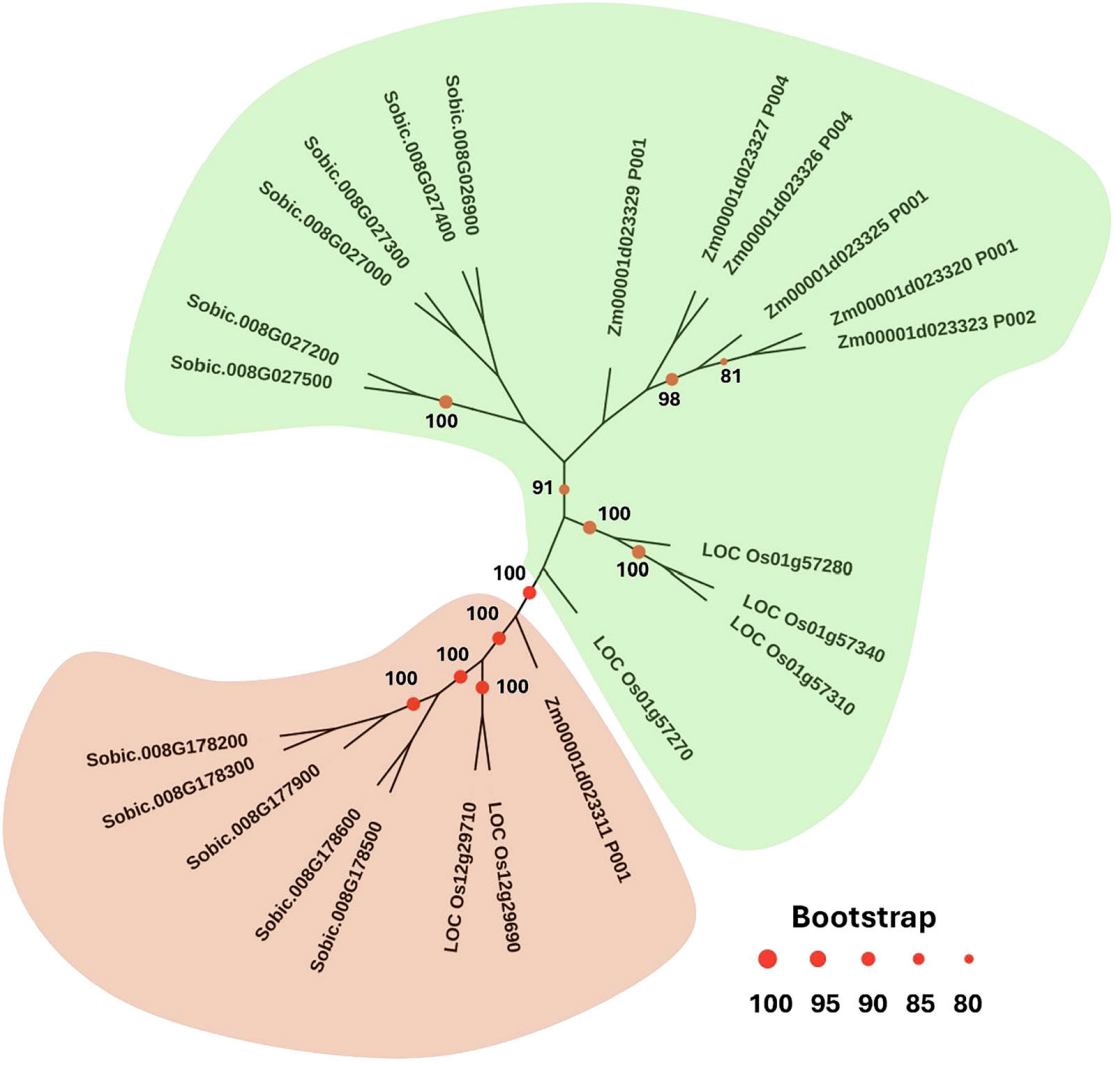
Maximum‐likelihood tree with 1000 bootstrap replicates among the sorghum, maize, and rice *Rp1‐D* homologues (green clade) and *Rp2* homologous (brown clade) genes. Bootstrap values > 80 are marked on each node with red circle.

The comparative analysis of *Rp2* identified the presence of two orthologs genes in rice chromosome 12 (*LocOs12G29960* and *LocOs12g29710*) and one orthologs gene on maize chromosome 10 (*Zm00001d023311 P001*) (Figure 4). Phylogenetic analysis of these genes with the known (previously identified; Ramakrishna, et al. 2002) *Rp1* orthologs of maize (six genes), sorghum (four genes), and rice (six genes) showed that the *Rp2* locus clustered separately and must be considered a novel locus for rust resistance.

## 4 DISCUSSION

The genetic diversity of breeding programs in the United States relies in the NPGS tropical sorghum germplasm. Nevertheless, the integration and use of this genetic diversity in breeding programs has been limited by the size of the collection and the photoperiod sensitivity of tropical germplasm. The discovery and genomic dissections of agronomically important alleles in tropical germplasm could hasten the introgression process into elite varieties. The NPGS Sudan core collection is an important genetic diversity resource that includes landraces with valuable agronomic traits (Cuevas & Prom, 2020), superior grain quality (Cuevas & Prom, 2024), and resistant to anthracnose (Cuevas & Prom, 2020) and rust. Consequently, these Sudanese superior landraces could be used to increase sources of disease resistant and to expand the genetic diversity of breeding programs.

The frequency of rust‐resistant accessions in NPGS Sudan core (7%) was lower than that observed for anthracnose (*Colletotrichum sublineola*) resistant (21%; Cuevas & Prom, 2020). These foliar fungal diseases have different dispersion methods and life cycles due to higher aggressiveness of anthracnose compared to rust. The dispersion of *P. purpurea* in the field is influenced by the temperature, exposure to full sunlight, relative humidity, and length of leaf wetness period (White et al., 2014). In addition, this obligate fungal pathogen needs the presence of a close alternate host to initiate new infections on sorghum in the field (Little & Perumal, 2019). In contrast, anthracnose sclerotia, acervuli, and mycelia remain in soil and plants debris as inoculum between growing seasons (Stutts & Vermerris, 2020). Therefore, selection pressure for anthracnose resistant is likely to be higher than rust. In fact, a signature of selection for rust and anthracnose resistant was observed in population 3, but the total number of accessions resistant to rust was lower than that observed for anthracnose. We also observed that three accessions (PI 568557, PI 568621, and PI 570548) belonging to population 3 and another six admixture accessions (PI 562941, PI 568398, PI 56993, PI 570213, PI 570705, and PI 570708) were resistant to both diseases. Certainly, the origin of population 3 might be associated to the low‐rainfall savannah zone and the flood plain regions where sorghum is one of the main crops (Mahgoub, 2014). Moreover, these accessions are likely to capture a broad spectrum of resistant against other fungal diseases and could be used as parental lines in breeding programs.

Resistance loci in plants are mainly composed of clusters of several homologous R genes (Ellis et al., 2000a). Synteny analysis between the rust resistance loci *Rp1* and *Rph* in maize and sorghum, respectively, found that these orthologs loci are aggregates of six and five homologous R genes (Ramakrishna et al., 2002). The NBS–LRR encoding genes are arranged as clustered loci as a result of tandem duplication that led to a diversification of function (Leister, 2004). Therefore, the five homologous R genes in the *Rp2* locus might act together as one locus for the detection of different *P. purpurea* effectors or pathotypes. Thus, it is expected that the genetic diversity found in *Rp2* locus provides a broader spectrum of resistance against pathotypes found in other environments. Moreover, exon 1 of *Rp2* locus includes the NBS domain that mediates receptor oligomerization upon activation and can also participate in ligand binding (Prigozhin & Krasileva, 2021). The high number of SNPs in exon 1 that resulted in seven haplotypes also indicates this locus is evolving faster to overcome the pathogen diversity. The high frequency of recombination among R genes results in their rapid evolution and diversification (Chen et al., 2010). Our LD analysis found this cluster of five R genes is in a genomic region with high recombination rate, which has LD decay of 1.47 kbp. The functional genomic analysis of the *Rp2* locus against diverse pathotypes of *P. purpurea* could be used to elucidate the plant–pathogen interaction that led to a resistant response.

Comparative genome analysis against other rust resistance loci in sorghum (Colkman & Dean, 1961; Mace et al., 2009; McIntyre et al., 2005; Murali Mohan et al., 2010; Upadhyaya et al., 2013; X. Wang et al., 2014) found that *Rp2* locus is a novel resistance source in sorghum. The *Pu* and *Rp1‐D* loci and other QTL identified through linkage mapping and GWAS are in chromosome 8. However, the nearest QTL was QRustR_S2_8.2 (X. Wang et al., 2014) located downstream of *Rp2* locus. The *Pu* and *Rp1‐D* loci both are located at the top of chromosome 8 (Mace & Jordan, 2010); therefore, the stacking of multiple rust resistance loci into one common elite germplasm could be achieved through marker‐assisted selection. The analysis also found that the orthologs of *Rp2* locus in maize (*Zm00001d023311*) are associated with the resistant response to *Puccinia polysora*, the causal agent of southern corn rust (Lv et al., 2021). Likewise, gene expression analysis in rice after the infestation of the brown planthopper (*Nilaparvata lugens*) found that the expression of *Os12G29690* is upregulated in the resistant line (Guo et al., 2014). Grass genome synteny analysis between rice, sorghum, and maize found that the genomic block enclosing the *Rp2* locus evolved from a common ancestor (Zhang et al., 2014). Therefore, the results provide evidence that the R genes in the *Rp2* locus diversified to provide resistant against different pathogens and pests. The length of the LRR domain is an important contributor to R gene diversification (Ellis et al., 2000b). Therefore, it is possible that the *Rp2* orthologs region in other grasses such as wheat, sugarcane, and *Brachypodium* might also be associated with diseases‐resistant responses.

The introgression of *Rp2* locus into temperate‐adapted germplasm is the first step for its use in sorghum breeding programs. In this regard, the high recombination rate within the *Rp2* locus makes the development of several high‐throughput SNP markers across the 57 kbp genomic region necessary to introgress the five R genes. The SAP represents the genetic diversity of US breeding programs (Morris et al., 2013), but the rust resistance response of most of these lines is unknown. To get insight of the possible presence of *Rp2*‐resistance allele in the SAP, we evaluated the allele frequency of the SNP S8_61201912. The analysis found that the resistance allele of *Rp2* is rare in the SAP (minor allele frequency = 0.05) and could not be associated with Sudanese germplasm. In fact, the resistance alleles were found in sweet sorghum germplasm, elite breeding materials, and few converted lines from South Africa, Zimbabwe, Ethiopia, and Egypt. Furthermore, the confirmation of *Rp2* resistance allele in temperate‐adapted germplasm may require the development of biparental mapping populations. In this regard, the adaptation to temperate regions of the four most rust‐resistant Sudanese tropical accessions (PI 568621, PI 569393, PI 570548, and PI 570974) will provide new sources of rust resistant to breeding programs.

## AUTHOR CONTRIBUTIONS

**Hugo E. Cuevas**: Conceptualization; data curation; formal analysis; methodology; project administration; writing—original draft. **Louis K. Prom**: Conceptualization; formal analysis; methodology; writing—review and editing.

## DATA AVAILABILITY SATATEMENT

The phenotypic datasets generated during the current study are available in the supplementary table and through the US National Plant Germplasm System; GRIN‐Global.

## Supporting information




**Supplementary Table S1** Hierarchical organization of the genetic relatedness of 268 accessions from the USDA‐NPGS Sudan germplasm collection based on the population structure analysis of 5366 unlinked SNPs (Cuevas and Prom 2020) and rust resistant response.
**Supplementary Table S2**. Genetic variation and haplotypes found the exon 1 of the candidate rust resistance gene *Sobic.008G172800*.
